# Determinants of bed net use conditional on access in population surveys in Ghana

**DOI:** 10.1186/s12936-019-2700-7

**Published:** 2019-03-08

**Authors:** Emily Ricotta, Samuel Oppong, Joshua O. Yukich, Olivier J. T. Briët

**Affiliations:** 1 Swiss Tropical and Public Health Institute, Socinstrasse 57, P.O. Box CH-4002, Basel, Switzerland; 2 University of Basel, Petersplatz 1, P.O. Box CH-4001, Basel, Switzerland; 3 National Malaria Control Programme, Public Health Division, Ghana Health Service, Korle-bu, P. O. Box KB 493, Accra, Ghana; 4 Center for Applied Malaria Research and Evaluation, Tulane University School of Public Health and Tropical Medicine, 1440 Canal St. #8317, New Orleans, LA 70112 USA

**Keywords:** Malaria, Bed net, Remote sensing, Survey, Human behavior

## Abstract

**Background:**

Insecticide-treated nets (ITNs) are one of the most effective and widely available methods for preventing malaria, and there is interest in understanding the complexities of behavioural drivers of non-use among those with access. This analysis evaluated net use behaviour in Ghana by exploring how several household and environmental variables relate to use among Ghanaians with access to a net.

**Methods:**

Survey data from the Ghana 2014 Demographic and Health Survey and the 2016 Malaria Indicator Survey were used to calculate household members’ access to space under a net as well as the proportion of net use conditional on access (NUCA). Geospatial information on cluster location was obtained, as well as average humidex, a measure of how hot it feels, for the month each cluster was surveyed. The relationship between independent variables and net use was assessed via beta-binomial regression models that controlled for spatially correlated random effects using non-Gaussian kriging.

**Results:**

In both surveys, increasing wealth was associated with decreased net use among those with access in households when compared to the poorest category. In 2014, exposure to messages about bed net use for malaria prevention was associated with increased net use (OR 2.5, 95% CrI 1.5–4.2), as was living in a rural area in both 2014 (OR 2.5, 95% CrI 1.5–4.3) and 2016 (OR 1.6, 95% CrI 1.1–2.3). The number of nets per person was not associated with net use in either survey. Model fit was improved for both surveys by including a spatial random effect for cluster, demonstrating some spatial autocorrelation in the proportion of people using a net. Humidex, electricity in the household and IRS were not associated with NUCA.

**Conclusion:**

Net use conditional on access is affected by household characteristics and is also spatially-dependent in Ghana. Setting (whether the household was urban or rural) plays a role, with wealthier and more urban households less likely to use nets when they are available. It will likely be necessary in the future to focus on rural settings, urban settings, and wealth status independently, both to better understand predictors of household net use in these areas and to design more targeted interventions to ensure consistent use of vector control interventions that meet specific needs of the population.

**Electronic supplementary material:**

The online version of this article (10.1186/s12936-019-2700-7) contains supplementary material, which is available to authorized users.

## Background

Insecticide-treated nets (ITNs) are one of the most effective and available methods for preventing malaria, having averted an estimated 68% of malaria cases between 2000 and 2015 [1]. In 2007, the World Health Organization (WHO) recommended full coverage of ITNs for populations in areas at high risk for malaria transmission [2], which has been followed by a massive scaling up of ITN distribution programmes aimed at providing enough nets for all households. The success of these programmes has historically been evaluated by two primary indicators: the proportion of households owning at least one ITN, and the proportion of people using ITNs the night before the survey [3]. These indicators demonstrate that, on average since 2010, 54% of households in sub-Saharan Africa own at least one ITN, and 33% of the population reported using one the night prior to the interview [4]. However, these indicators do not capture whether a household has enough nets for all members and if members have access to a net, which is why new indicators were introduced measuring the proportion of households which have at least one net per two people, as well as the proportion of the population with ITN access [3]. Knowing what proportion of households have enough nets to cover all members is useful for net distribution campaign planning, and knowing population access provides a way to understand net use patterns in the population that has access [4, 5].

Out of 27 President’s Malaria Initiative (PMI) focus countries surveyed with a Demographic and Health Survey (DHS) or Malaria Indicator Survey (MIS) in the 2010–2017 period, Ghana ranks 25th in net use conditional on access (ratio use to access 0.63, 2016 MIS); only Niger (2012 DHS) and Zimbabwe (DHS 2015) have a larger proportion of the population not using nets despite having access to a space under one [4]. In Ghana, the proportion of the population with access to an ITN within their household varies widely by region and wealth quintile. Greater Accra seems to have a particularly large discrepancy between ITN use and access (use:access ratio 0.34); however, none of the regions of the country have a ratio above 0.8 [4].

While access to an ITN within the household is the best predictor of ITN use, given the differences in use conditional on access seen across Ghana it is important to understand what additional variables might explain use behaviour when household members theoretically have access to a space under a net within their household. Some self-reported reasons for not using nets in the literature include discomfort (due to heat) [6], fluidity of sleeping arrangements (i.e. moving from inside to outside or vice versa during the night) [7], and little perceived need to use a net when mosquito density is low [6, 8]. The goal of this analysis was to explore how several variables, including connection to electricity, exposure to net use messaging, and ‘humidex’ (how hot the weather feels to an average person), relate to net use among Ghanaians who theoretically had access to a space under a net in their household.

## Methods

Data from the Ghana 2014 Demographic and Health Survey (DHS) and the 2016 Malaria Indicator Survey (MIS) were obtained from the DHS Programme [9], analysed separately, and compared in this analysis. Geospatial information about each cluster of households (one geolocation per cluster), defined as a census enumeration area of either a village or an urban city block, was obtained as well. Cluster locations are offset between 0 and 2 km for urban clusters, and 0–5 km for rural clusters (with an additional 1% displaced up to 10 km) to retain confidentiality [10]. Access to a net was calculated in a different manner than recommended by the Roll Back Malaria Monitoring and Evaluation Reference Group Survey and Indicator Task Force [3], in which access is defined as the number of ITNs in the household multiplied by two, but capped by the number of people who stayed in the household. Instead, access to any net (ITNs and non-insecticidal nets) in a household was calculated as the sum of the number of people who slept under a net and the number of people who stayed the night and could have slept under a net yet did not, assuming two free spaces per unoccupied net and one free space per net that had a single occupant [11]. The proportion of household members that used a net out of those that had access, the ‘net use conditional on access’ (NUCA), was calculated as the number of household members that slept under a net divided by the number with access.

To understand discomfort in the use of nets due to heat, the humidex (a unitless index) was calculated from spatio-temporal data of temperature and vapour pressure from the CRU TS v. 4.01 dataset which has monthly temporal resolution on 0.5 × 0.5 degree grids) [12, 13] using the formula

$$Humidex = T +( 0. 5 5 5 5 { }* \, \left( {e - 10} \right))$$

where *T* is the temperature in degrees Celsius, and *e* is the vapour pressure in millibars [14]. These data were extracted at the latitude and longitude point for each cluster for the month and year in which the cluster was surveyed, using ArcGIS 10.5 [15]. Four clusters out of 427 in 2014 and eight out of 200 in 2016 were excluded due to missing coordinates.

Statistical analysis was done in R 3.4.2 [16]. The unit of analysis was households. Means and proportions were calculated using the “survey” package to account for sampling design by incorporating the DHS or MIS survey weights, as appropriate [17, 18]. Pearson’s Chi squared test statistic was used to evaluate differences in access, with a statistical significance level of 0.05. Using the R-INLA package [19–23], the relationship between independent variables and net use among those who had access to a space under a net the night before the survey in households was assessed. Beta-binomial regression models that controlled for spatially correlated random effects using non-Gaussian kriging were chosen to account for overdispersion in the data. By design, households without nets or without people spending the night at home were excluded from the analysis. Candidate explanatory variables for each model included region, setting (urban or rural), mean humidex for the cluster, wealth quintile [24], indoor residual spraying (IRS) of the dwelling with insecticides within the previous year, connection to electricity, ratio of nets to household members, and whether the respondent heard a message on the use of nets. Explanatory variables and first-order interaction terms were chosen via forward stepwise selection using the Deviance Information Criterion (DIC). Raster images of predicted NUCA and net access from INLA output with spatial effects only (i.e. no covariates) were created using the ‘rgdal’ and ‘raster’ packages and imported into ArcGIS to plot maps [25, 26]. The 2016 and 2014 maps were compared by examining the overlap of the 95% credible intervals of the logit transformed maps of both access and NUCA.

## Results

### Survey characteristics

Interviews for the 2014 DHS took place from September through December 2014. There were 11,835 heads of households interviewed, with these households comprising of 40,337 individual members, and with an average size of 3.5 members per household. Interviews for the 2016 MIS were conducted from October through December 2016. Over a 6-week period, heads of 5841 households were interviewed, with 20,708 individual household members, and with an average household size of 3.6. Forty-three percent of individuals were < 15 years old in both surveys. For both the 2014 DHS and 2016 MIS, 55% of the households were classified as urban and 45% as rural. The median humidex during the months of interview in 2014 was 40 (interquartile range [IQR]: 36), and was 35 (IQR: 33) in 2016 (Additional file 2: Table S1).

### Net ownership and use

**Fig. 1:**
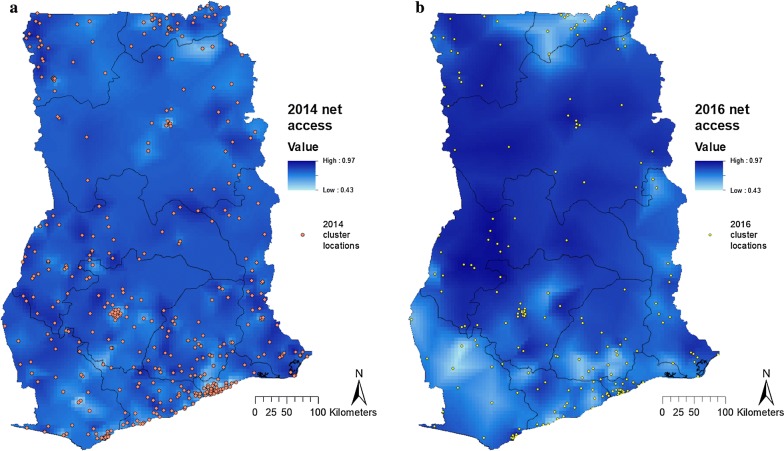
Net access in Ghana in the **a** 2014 DHS, and **b** 2016 MIS

In 2014, there were 16,892 nets in the surveyed households, 16,463 (97%) of which were ITNs. This proportion was similar in 2016, when there were 10,689 nets, 10,490 (98%) of which were ITNs. In 2014, households had an average of 1.4 [95% confidence interval (CI) 1.3–1.4] nets, which increased to 1.7 (95% CI 1.6–1.7) nets in 2016. The proportion of households without any net decreased from 30% (95% CI 29–32) to 26% (95% CI 24–28); access increased from 63% of the population (95% CI 62–65) having access to a sleeping space under a net in 2014, to 70% (95% CI 68–72) in 2016. Mean net access over all households within a cluster varied across the country (Fig. 1). In 2014, the cluster level access to a net ranged from 18 to 100%, and in 2016, it varied from 37 to 100%. The proportion of individuals with theoretical access was significantly lower in urban areas than in rural areas in both 2014 (57% of household members with theoretical access in urban vs 73% in rural areas, p < 0.001) and 2016 (61% in urban *vs* 80% in rural areas, p < 0.001).

**Fig. 2:**
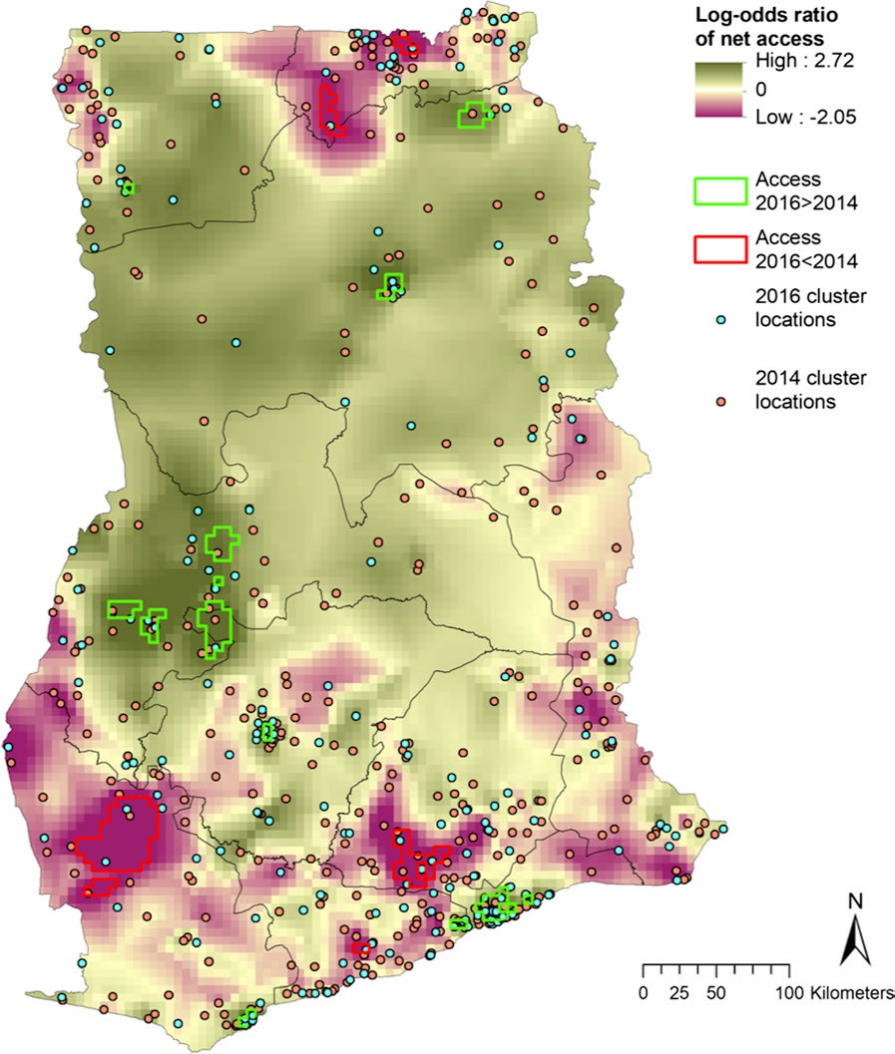
Log odds ratio of NUCA in 2016 relative to 2014. Areas where the lower 95% credible boundary of 2016 was above the upper boundary of 2014 access are demarcated in green, and areas where the lower 95% credible boundary of 2014 access was above the upper boundary of 2016 access are demarcated in red

**Fig. 3:**
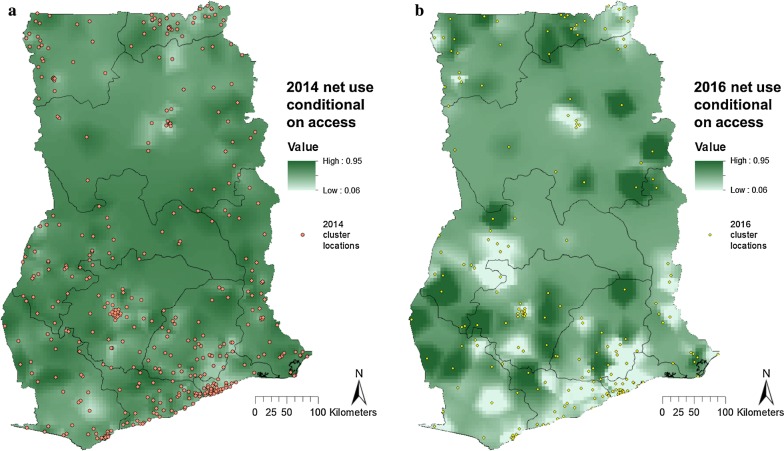
Net use conditional on access in Ghana in the **a** 2014 DHS, and **b** 2016 MIS

Access in 2016 was significantly higher than 2014 in parts of western Brong Ahafo, Accra, central and north Northern region and a few other sporadic areas (Fig. 2, green). Net access in 2016 was significantly lower than 2014 in parts of the Western, Eastern, and Upper East regions (Fig. 2, red). Mean NUCA in 2014 was 0.52 (95% CI 0.50–0.54), and this increased in 2016 to a NUCA of 0.55 (95% CI 0.52–0.59). Clustering of the NUCA was observed in both surveys (Fig. 3). Overdispersion of net use can be seen in Additional file 1: Fig. S1, where the majority of households had either all members under a net, or no-one using a net. The proportion of nets that were unoccupied, were occupied by only one person, by two people, etc. was similar for both surveys (Additional file 1: Fig. S2).

### Net use conditional on access

**Fig. 4:**
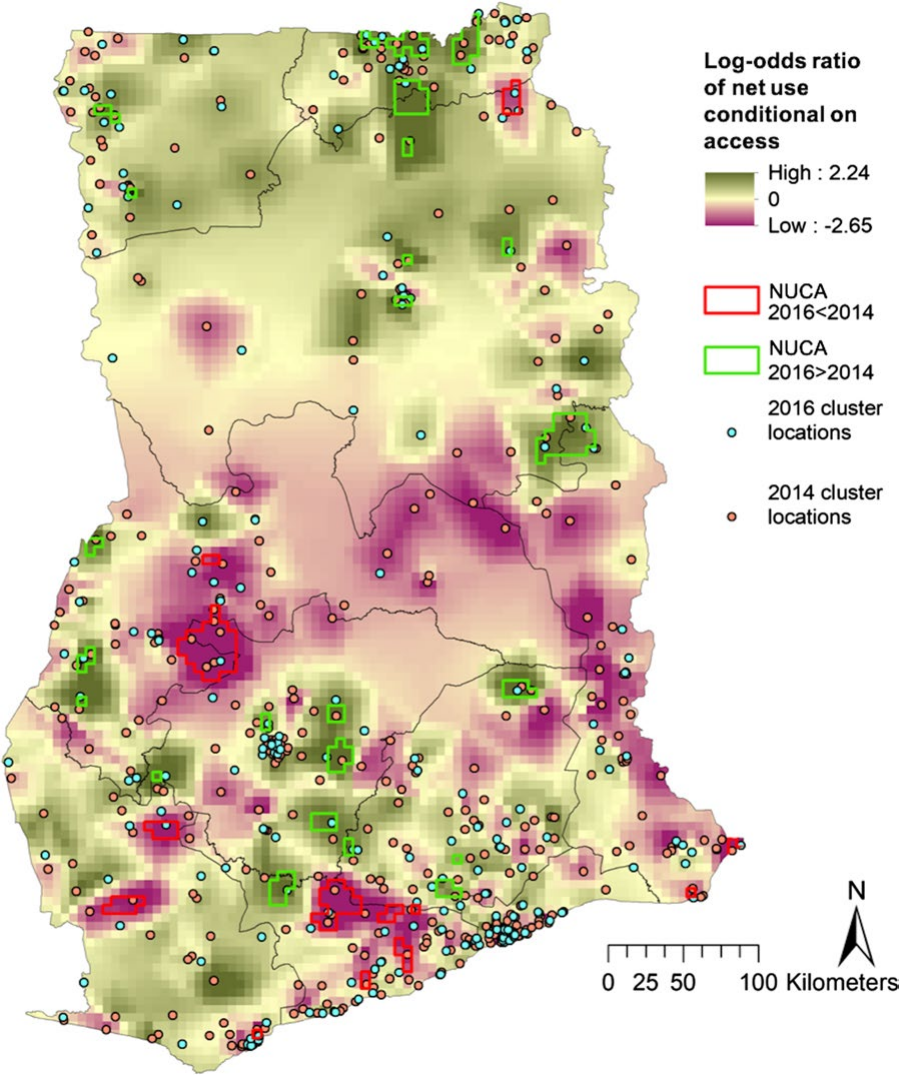
Log odds ratio of net use conditional on access in 2016 relative to 2014. Areas where the lower 95% credible boundary of 2016 was above the upper boundary of 2014 access are demarcated in green, and areas where the lower 95% credible boundary of 2014 access was above the upper boundary of 2016 access are demarcated in red

As the analysis of net use was conditional on households having (some) access to nets, households without any nets were excluded from this analysis. This left 8385 out of 11,835 households (71%) in 2014 and 4279 households out of 5841 (73%) in 2016 (this increase was statistically significant, p < 0.001). Comparing the NUCA without accounting for covariates in 2016 to that in 2014, at least some significant increase in NUCA was seen in most regions in the country (Fig. 4, green). A significantly decreased NUCA was seen primarily in the south of the country, although there was also a significant reduction in a small section in the northernmost part of the Northern region (Fig. 4, red).

The final, selected, models for both the 2014 DHS and the 2016 MIS included setting (urban or rural), wealth quintile, IRS of the dwelling with insecticides in the year prior to the survey, the ratio of nets to household members, the humidex in the month of survey, connection to electricity, and whether the respondent heard a message on the use of nets. Interaction terms between messaging, setting, IRS, and electricity were included to improve model fit but were not significantly associated with net use conditional on access.

In both surveys, increasing wealth was associated with decreased net use among those with theoretical access to a net in households when compared to the poorest category (Table 1). In 2014, having heard a message about using a bed net for malaria prevention was associated with increased net use [odds ratio (OR): 2.5, 95% credible interval (CrI) 1.5–4.2]. Living in a rural area was also significantly associated with an increase in net use in both 2014 (OR 2.5, 95% CrI 1.5–4.3) and 2016 (OR 1.6, 95% CrI 1.1–2.3). Model fit was improved for both surveys with the inclusion of a spatial random effect for cluster as evidenced by a reduced DIC (18,439.56 to 13,215.01 in 2014 and 7501.88 to 7227.06 in 2016), demonstrating that there is some spatial autocorrelation in NUCA (Additional file 2: Table S2). Coefficients of the humidex in the month of survey, electricity in the household, and having received IRS in the last 12 months were not significantly different from zero.

**Table 1:** Regression coefficients and 95% credible intervals for explanatory variables of net use conditional on access in betabinomial regression models with spatial random effects

Effect	2014		2016	
	Exponentiated coefficient	95% credible interval	Exponentiated coefficient	95% credible interval
Wealth (ref: poorest)				
Poorer	0.99	(0.82–1.20)	0.94	(0.74–1.20)
Middle	0.77*	(0.62–0.96)*	0.69*	(0.52–0.91)*
Richer	0.55*	(0.43–0.71)*	0.60*	(0.45–0.81)*
Richest	0.48*	(0.36–0.64)*	0.44*	(0.32–0.61)*
Number of nets per person	1.02	(0.89–1.16)	1.07	(0.90–1.27)
Humidex	1.00	(0.99–1.00)	1.00	(0.99–1.01)
Messaging	2.53*	(1.52–4.22)*	1.16	(0.75–1.83)
Setting (ref: urban)	2.54*	(1.51–4.31)*	1.59*	(1.08–2.33)*
IRS in last 12 months	0.76	(0.36–1.59)	0.82	(0.45–1.50)
Electricity in household	0.84	(0.52–1.36)	0.76	(0.53–1.08)
Messaging × rural	0.68	(0.42–1.10)	1.19	(0.87–1.65)
Messaging × IRS	0.76	(0.42–1.36)	1.12	(0.72–1.75)
Messaging × electricity	0.94	(0.60–1.47)	1.03	(0.68–1.57)
Rural × IRS	1.15	(0.76–1.74)	1.09	(0.67–1.76)
Rural × electricity	0.79	(0.58–1.07)	0.85	(0.57–1.26)
IRS × electricity	1.06	(0.74–1.52)	0.77	(0.47–1.25)

× Indicates interaction term

*Statistically significant (p ≤ 0.05)

## Discussion

This analysis evaluated net use conditional on access to a bed net in Ghana by incorporating household-level predictors of net use and geographic aspects such as location and humidex. As Ghana’s NUCA is low throughout the country, especially in urban areas, relative to other countries, exploring associations with other factors may improve insight into what actions could be taken to increase use of nets by those that have access.

There has been a lot of interest in understanding the complexities of behavioural drivers of non-use among those with access [6, 27, 28]. These complexities include issues surrounding personal comfort or convenience of using nets, beliefs about personal risk of disease, and the feelings of one’s community and social network toward nets and their value [29–32]. The interplay of these factors is important, as it can help us understand how these variables are influenced by community engagement and campaigns aimed at education and behaviour change [29].

While there are many reasons people cite for non-use [8, 33, 34], thermal discomfort when using a net is common [8, 35, 36]. Indeed, in rural areas in northern Ghana, the practice of sleeping outside during the dry season for the fresher, cooler air is widespread [7]. Because of this, the humidex, which combines temperature and vapour pressure into a measure of how hot the weather felt during the month in which the survey took place, was included in this analysis. While the inclusion of this variable improved the fit of our models for both 2014 and 2016, in the final adjusted model, the humidex did not have a significant association with net use. Studies have shown that bed nets decrease airflow [37], and it is likely that this is what makes it feel hot and stifling under a net [38–40]. A pilot study has been conducted on attempting to improve comfort under nets by including small fans meant to increase air flow [41], but further research is needed to understand whether this would increase net use.

Access was correlated with setting, with rural households having consistently better access to bed nets than urban households. Due to lower disease burden in the urban areas, which make up 55% of Ghana’s population, cities such as Accra were deprioritized by malaria control campaigns [42]; however, a national ITN distribution campaign was conducted in 2014–2015, and in May 2016 there was additionally a country-wide scale up of school-based continuous distribution in an attempt to decrease gaps in net access across the country [43, 44]. Indeed, the proportion of households with enough nets for all household members was significantly higher in 2016 in both rural (65% in 2016 vs 58% in 2014, p < 0.001) and urban (50% vs 46%, p = 0.001) settings than in 2014.

Net use conditional on access was also spatially correlated; inclusion of a spatial random effect in the regression models for use conditional on access significantly improved the fit. In addition to having better access to nets, rural households were more likely to use them in 2014. This has been observed in Ghana for a number of years [33, 45]. Among households with access, one possible reason for non-use among the urban population is a lower perceived risk of disease from malaria, especially as disease prevalence is much lower in urban areas than rural ones [46, 47]. This is attributed mainly to two things: first, decreases in breeding sites and resting places for mosquitoes, partly attributable to source management and larviciding which has severely diminished the mosquito population, and second, urban households in Ghana have used alternative protective measures such as house screening (over 80% of the urban households in Accra have window screens), aerosol sprays, and mosquito coils for several years [48].

Additionally, it has been shown that both perceptions of susceptibility to and severity of malaria have been associated with increases in net usage, while the belief in one’s ability to detect and treat a case of malaria has shown a negative correlation with net use [27, 29]. It is possible that urban populations who are at decreased risk of malaria are less worried about (severe) disease, or feel that they are better able to manage the illness if it does occur, thus lessening their belief in the benefit of using a net [47, 49]. Whatever the reason for non-use among those with access in urban settings, it is likely different enough from rural communities that targeting alternative interventions or educational messaging based on setting might be warranted. It will also be important to continue to monitor risk of malaria in urban communities, to ensure that the rapid urbanization and subsequent changes in infrastructure do not re-introduce malaria into areas where control is minimal.

Finally, multiple studies have shown a positive relationship between behaviour change communication (BCC) and net use in several countries [27, 50–53]. Ghana has a long history of using mass media and other communication channels to educate the population about malaria, and knowledge about transmission and prevention is generally good [54]. In the first half of 2014, the national communication strategy was revised to reflect updated WHO recommendations and NMCP policy. Special focus was placed on increasing advocacy, communication, and social mobilization for a number of malaria-related topics, including LLIN use and maintenance, and both advertising agencies and other health communication partners were engaged to produce educational materials. In the first quarter of 2016 alone, there were a reported 41 radio and television programmes in both English and local languages dedicated to educating the public on malaria control interventions [55]. These campaigns appear to have been moderately successful, because there was a significant relationship between messaging exposure and net use observed in the 2014 survey, with individuals who were exposed to messaging being 2.5 times more likely to use a net than those without message exposure. What is unclear is why the same relationship was not seen in 2016. In the 2014 DHS, exposure to malaria messaging was remarkably high, with 92% of respondents recalling any exposure to messaging. Messaging included appropriate care-seeking behaviour as well as use of ITNs by families, specifically pregnant women and children. More men than women had access to mass media (86% vs 69%), and it is conceivable that because in many regions of Ghana, the male head of household is responsible for decision making about health topics, their greater exposure to messaging leads to a larger observed effect in net use. In the 2016 MIS, only 46% of women reported having been exposed to general malaria messaging (as MISs focus on specific target populations, men were not interviewed and their level of exposure to messaging is unknown). This evidence, coupled with the significant decrease seen in NUCA in some regions of Ghana between 2014 and 2016 (Fig. 4), suggests a need for increased net use BCC.

Exposure to messaging is generally higher in urban areas than rural ones. Additionally, urban residents are more likely to encounter messages via mass media such as radio and TV, while rural residents are more frequently exposed to messaging at health centers and from community health workers. However, according to a health communication survey conducted in 2015, health workers were the most trusted source of information [56]. This might be one contributing factor to why net use in rural communities is higher; even though urban residents might be hearing a larger quantity of messages, they might consider them less trustworthy, which would lessen their impact on use. This phenomenon was demonstrated by Owusu Adjah and Panayiotou, who found that hearing messaging from a health worker had the highest adjusted odds of net use out of any of the measured messaging channels [52]. This highlights another important difference between urban and rural areas for malaria control, and emphasizes the need for unique targeting of interventions as well as messaging and education to each setting.

While, theoretically, everyone in the household has (some degree of) access to available net space, in reality, access of members in the household is determined by a number of factors including the household composition (e.g. the number of children), sleeping spaces, and other household arrangements [57–59]. In that light, it is somewhat surprising that the NUCA was not associated with the ratio of nets to household members (Additional file 1: Fig. S1), as one might expect that access (and hence use conditional on theoretical access) might increase with an increasing ratio of nets to household members until the point where everyone in the household has access to his/her own net. Once there are enough nets available in the household, supplying more nets will not increase net use. Also, the overdispersion of household NUCA data in this study (most households having either the maximum number of members using the available net space, or no members using available nets) implies that the decision about net use is made at the household level (e.g. by the head of the household or primary caretaker) rather than by each member independently. A more detailed understanding of why these differences exist among households that have access to nets might allow developing targeted strategies to improve net use.

This study has some limitations. One limitation is that both surveys were carried out over the same months of the year, and while this provides the benefit of consistency, it leaves an incomplete picture of net use behaviour for the remainder of the year. This is important because both surveys were conducted during the dry season, and net use has been shown to vary by season [60]. It is possible that at a different time of the year, or in temporal studies, humidex would become a significant predictor of net use among those with access. As both surveys were cross-sectional and conducted independently, they did not capture the exact same households.

Also, while studies using large-scale population surveys can find associations between household and population factors and net use, actually understanding the nuance of these associations requires in-depth analysis at the local level. For example, while this study found a negative association between wealth status and net use, it does not allow understanding why and how wealth status influences net use. This could be related to household setup, sleeping space designation and allotment within the family, or particular sources of income. Wealthier households tend to have better access to housing improvements like window screens and closed eaves that reduce exposure to mosquito bites indoors [61]. Along with this, having a decreased perception of vulnerability to malaria has been shown to decrease net use [62]. A study by Galactionova et al. [63] found that net ownership and use vary widely across sociodemographic characteristics within and across countries. In addition, spatiotemporal patterns in net use that are not explained by predictors such as household setting or wealth could be due to cultural or religious influences that are not adequately captured in population surveys.

## Conclusions

While household net use conditional on access is low in Ghana, it is spatially-dependent. Setting (whether the household was urban or rural) also plays a role, with wealthier and more urban households less likely to use nets when they are available. It will likely be necessary in the future to focus on rural settings and urban settings and wealth status independently, both to better understand predictors of net use at the household level in these areas and to design more targeted interventions to ensure consistent use of vector control methods by the entire population.

## Additional files

**Additional file 1: Figure S1.** Net use in Ghana depending on ITN ownership in 2014 and 2016. **Figure S2.** Proportion of nets by number of people occupying them the night before the survey.

**Additional file 2: Table S1.** Table of summary survey statistics. **Table S2.** Regression coefficients and Deviance Information Criterion for models with and without a spatial random effects.
