# Supplementary material for: Determinants of bed net use conditional on access in population surveys in Ghana
Source: Malar J. 2019 Mar 8;18:63. doi: 10.1186/s12936-019-2700-7 (PMC6408824; doi:10.1186/s12936-019-2700-7)

Additional file 1 (PDF):

Supplemental Figure 1. Net use in Ghana depending on ITN ownership. A 2014; B 2016. The size of the circle corresponds to the number of households with that value. The green line shows theoretical optimum net use at one net per two people (universal coverage). The red line shows what could be expected if no one shared a net. The gray line is the fitted value using a beta-binomial regression model with logit-link function.


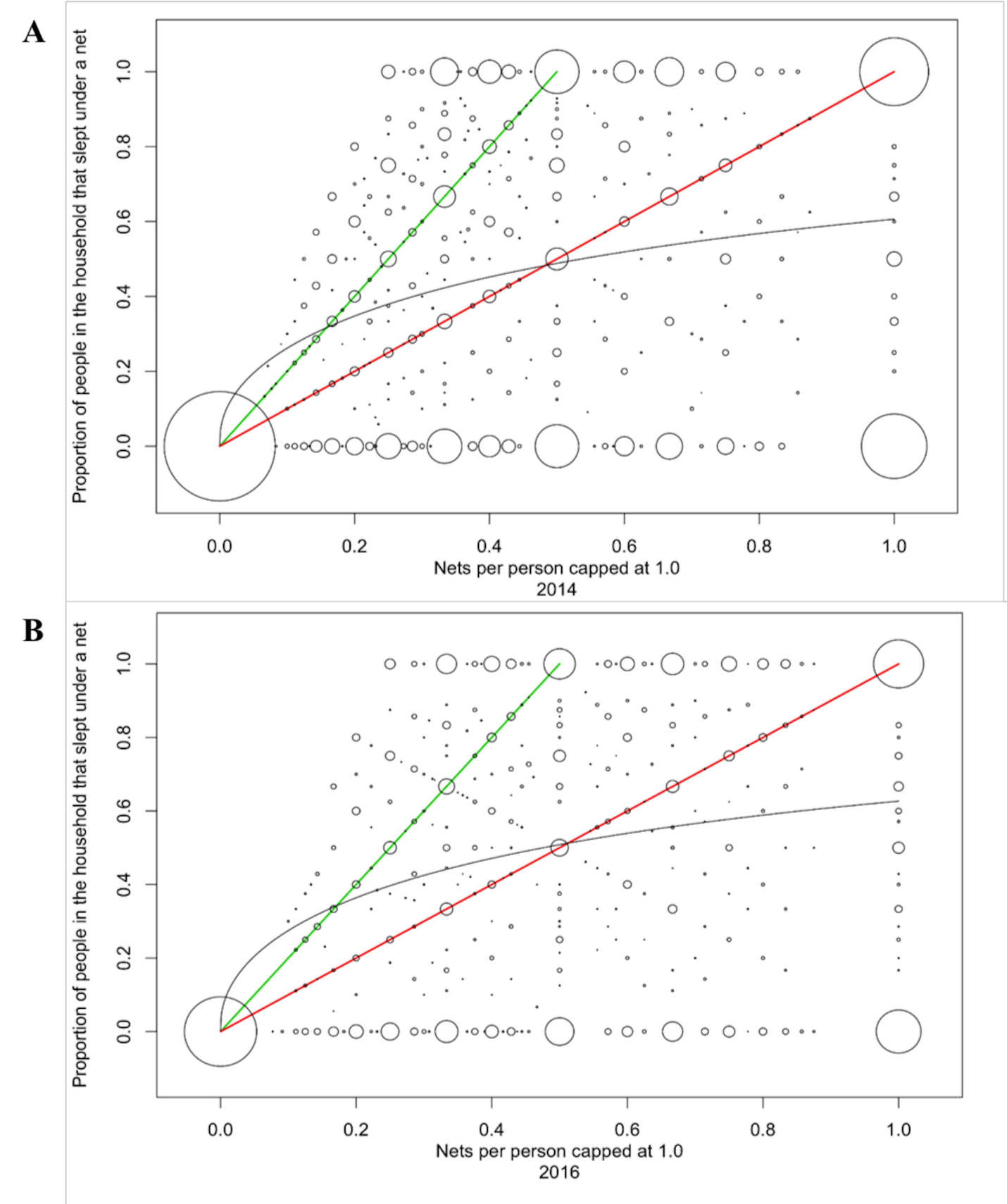


Supplemental Figure 2. Proportion of nets by number of people occupying them the night before the survey


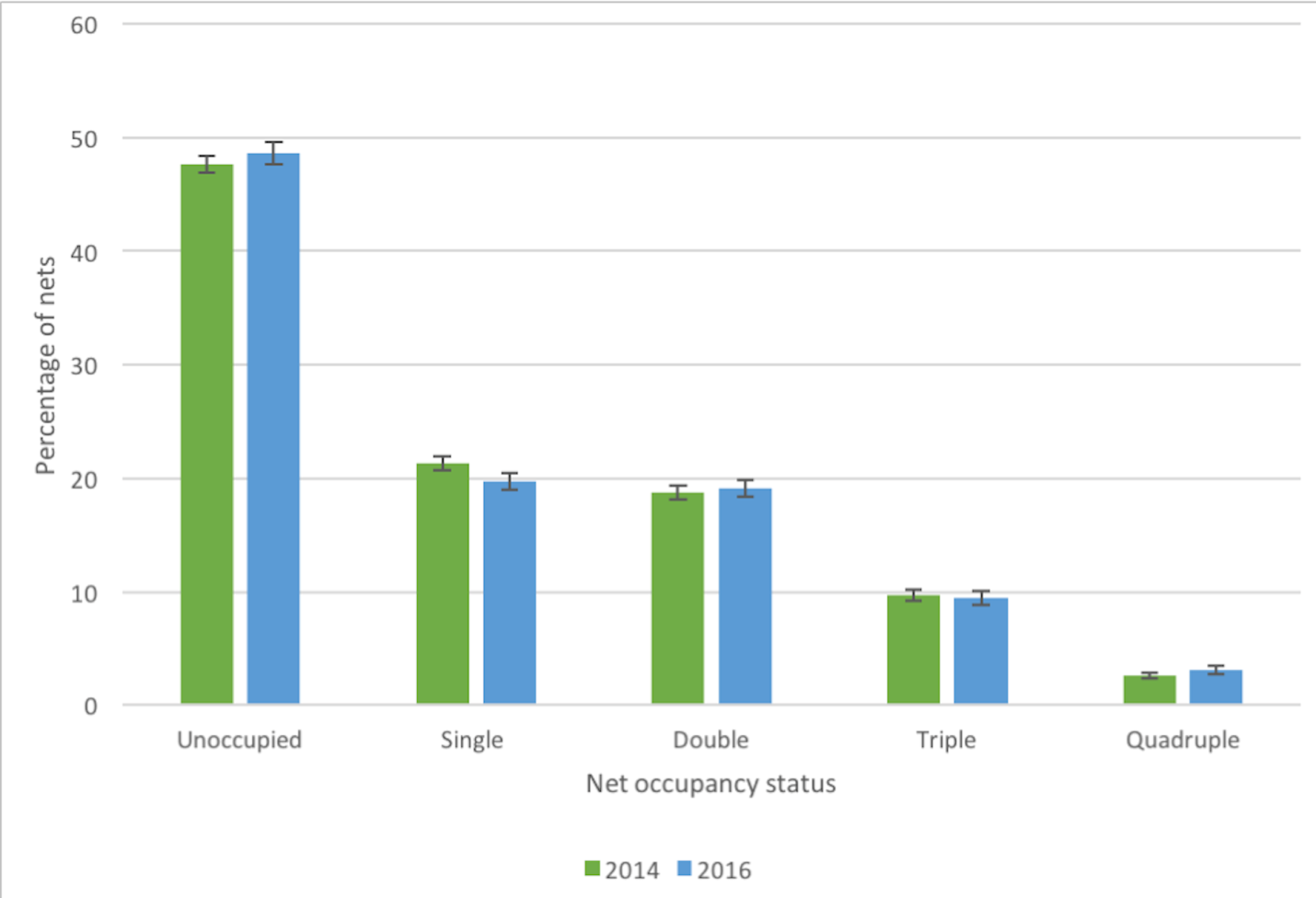

Supplement: Supplementary file 1 — Additional file 1: Figure S1. Net use in Ghana depending on ITN ownership in 2014 and 2016. Figure S2. Proportion of nets by number of people occupying them the night before the survey. [file 12936_2019_2700_MOESM1_ESM.docx]
