# Supplementary material for: Determinants of bed net use conditional on access in population surveys in Ghana
Source: Malar J. 2019 Mar 8;18:63. doi: 10.1186/s12936-019-2700-7 (PMC6408824; doi:10.1186/s12936-019-2700-7)
Supplement: Supplementary file 2 — Additional file 2: Table S1. Table of summary survey statistics. Table S2. Regression coefficients and Deviance Information Criterion for models with and without a spatial random effects. [file 12936_2019_2700_MOESM2_ESM.docx]

Additional Table S1: Summary survey statistics

| Survey | 2014 DHS | 2016 MIS |
| --- | --- | --- |
| Time period | Sep.–Dec. 2014 | Oct.–Dec. 2016 |
| Number of households interviewed | 11,835 | 5,841 |
| Number of household members in interviewed households | 40,337 | 20,708 |
| Members per household | 3.5 | 3.6 |
| Percent of household members <15 years old | 43 | 43 |
| Percent of households classified as urban | 55 | 55 |
| The median humidex during the months of interview | 40 | 45 |
| The IQR humidex during the months of interview | 36 | 33 |
| Households with one or more nets | 8,385 | 4,279 |
| Number of nets in surveyed households | 16,892 | 10,689 |
| Number of ITNs in surveyed households | 16,463 | 10,490 |
| Range in cluster level access to nets | 18%–100% | 37%–100%. |
| Percent of members with theoretical access in urban households | 57 | 61 |
| Percent of members with theoretical access in rural households | 73 | 80 |
| Nets per household in urban areas | 1.7 | 2 |
| Nets per household in rural areas | 2.7 | 3.1 |
| NUCA | 0.52 (95% CI: 0.50–0.54) | 0.55 (95% CI: 0.52–0.59) |

Additional Table S2. Regression coefficients with and without spatial random effects

| A | 2014 | | | | | |
| --- | --- | --- | --- | --- | --- | --- |
|  | With spatial random effects | | | Without spatial random effects | | |
|  | mean | 0.025 quantile | 0.975 quantile | mean | 0.025 quantile | 0.975 quantile |
| Poorer | 0.99 | 0.82 | 1.20 | 1.11 | 0.97 | 1.27 |
| Middle | 0.77 | 0.62 | 0.96 | 0.81 | 0.69 | 0.94 |
| Richer | 0.55 | 0.43 | 0.70 | 0.52 | 0.44 | 0.62 |
| Richest | 0.48 | 0.36 | 0.64 | 0.36 | 0.29 | 0.44 |
| Number of nets per person | 1.02 | 0.89 | 1.16 | 1.18 | 1.07 | 1.30 |
| Humidex | 1.00 | 0.99 | 1.00 | 1.00 | 1.00 | 1.00 |
| Messaging | 2.53 | 1.52 | 4.22 | 1.79 | 1.18 | 2.70 |
| Setting | 2.54 | 1.51 | 4.31 | 2.19 | 1.47 | 3.28 |
| IRS in last 12 months | 0.76 | 0.36 | 1.60 | 0.75 | 0.47 | 1.20 |
| Electricity in household | 0.84 | 0.52 | 1.36 | 0.82 | 0.55 | 1.22 |
| Messaging x rural | 0.68 | 0.42 | 1.10 | 0.87 | 0.59 | 1.27 |
| Messaging x IRS | 0.76 | 0.42 | 1.36 | 0.67 | 0.44 | 1.02 |
| Messaging x electricity | 0.94 | 0.60 | 1.47 | 1.01 | 0.70 | 1.45 |
| Rural x IRS | 1.15 | 0.76 | 1.74 | 0.95 | 0.75 | 1.21 |
| Rural x electricity | 0.79 | 0.58 | 1.07 | 0.78 | 0.62 | 0.98 |
| IRS x electricity | 1.06 | 0.74 | 1.52 | 1.20 | 0.95 | 1.51 |
|  | DIC: 13215.01 |  |  | DIC: 18439.56 |  |  |

| B | 2016 | | | | | |
| --- | --- | --- | --- | --- | --- | --- |
|  | With spatial random effects | | | Without spatial random effects | | |
|  | mean | 0.025quant | 0.975quant | mean | 0.025quant | 0.975quant |
| Poorer | 0.94 | 0.74 | 1.20 | 0.99 | 0.80 | 1.22 |
| Middle | 0.69 | 0.52 | 0.91 | 0.69 | 0.54 | 0.87 |
| Richer | 0.60 | 0.44 | 0.81 | 0.58 | 0.45 | 0.75 |
| Richest | 0.44 | 0.32 | 0.61 | 0.37 | 0.29 | 0.49 |
| Number of nets per person | 1.07 | 0.90 | 1.27 | 1.08 | 0.92 | 1.27 |
| Humidex | 1.00 | 0.99 | 1.01 | 0.99 | 0.99 | 1.00 |
| Messaging | 1.16 | 0.75 | 1.83 | 0.94 | 0.61 | 1.44 |
| Setting | 1.59 | 1.08 | 2.33 | 1.94 | 1.39 | 2.71 |
| IRS in last 12 months | 0.82 | 0.45 | 1.50 | 0.62 | 0.38 | 1.01 |
| Electricity in household | 0.76 | 0.53 | 1.08 | 0.75 | 0.54 | 1.04 |
| Messaging x rural | 1.19 | 0.87 | 1.65 | 1.20 | 0.88 | 1.63 |
| Messaging x IRS | 1.12 | 0.72 | 1.75 | 1.22 | 0.80 | 1.86 |
| Messaging x electricity | 1.03 | 0.68 | 1.57 | 1.12 | 0.75 | 1.67 |
| Rural x IRS | 1.09 | 0.67 | 1.76 | 0.94 | 0.63 | 1.42 |
| Rural x electricity | 0.85 | 0.57 | 1.26 | 0.76 | 0.53 | 1.08 |
| IRS x electricity | 0.77 | 0.47 | 1.25 | 1.09 | 0.72 | 1.65 |
|  | DIC: 7227.06 |  |  | DIC: 7501.88 |  |  |
